# Not evolved to save the planet, yet capable to promote pro-environmental action leveraging human nature

**DOI:** 10.3389/fpsyg.2025.1571765

**Published:** 2025-07-16

**Authors:** Marco Antonio Correa Varella, Felipe Carvalho Novaes, Ramon Felipe Bicudo da Silva, Renato de Mei Romero, Paulo Henrique Santos Gonçalves, Joelson Moreno Brito de Moura, Risoneide Henriques da Silva, Matheus Adriano Ferreira Coelho, João Vitor Rodrigues Costa, Mauro Dias Silva Júnior, Markus J. Rantala, Katariina Elsa Maria Vuorinen

**Affiliations:** ^1^Department of Botany, Center for Biosciences, Federal University of Pernambuco, Recife, Brazil; ^2^Department of Experimental Psychology, Institute of Psychology, University of São Paulo, São Paulo, Brazil; ^3^Resiclima Network, International Collaboration for the Multidimensional and Interdisciplinary Study of Global Climate Change, Recife, Brazil; ^4^Pontifícia Universidade Católica do Rio de Janeiro (PUC-Rio), Rio de Janeiro, Brazil; ^5^Laboratory of Spatial Analysis, Environmental Conservation, and Sustainability, Center for Environmental Studies and Research, State University of Campinas, Campinas, Brazil; ^6^Federal Institute of Education Science and Technology of Alagoas (IFAL), Campus Marechal Deodoro, Alagoas, Brazil; ^7^Instituto de Estudos do Xingu (IEX), Universidade Federal do Sul e Sudeste do Pará, São Félix do Xingu, Brazil; ^8^Evolution, Morality, and Politics Research Group, Universidade Federal Rural do Rio de Janeiro, Rio de Janeiro, Brazil; ^9^Behavioral Sciences Program, Department of Basic Psychological Processes, Institute of Psychology, University of Brasília, Brasília, Brazil; ^10^Department of Biology, University of Turku, Turku, Finland; ^11^Norwegian Institute for Nature Research (NINA), Lillehammer, Norway

**Keywords:** anthropogenic environmental problems, climate change, evolutionary psychology, pro-environmental behavior, environmental protection, behavioral science, evolutionary social sciences, sustainability

## Abstract

Anthropogenic environmental issues, from global warming to pollution, biodiversity loss, and natural resources depletion, require immediate action. Yet, inaction remains pervasive, and pro-environmental psychological interventions have, at best, yielded modest, short-lived effects. In this article, we argue that the development of more effective interventions could be aided by more nuanced discussion around two pervasive misguided assumptions: That human nature is inherently environmentally friendly, thus naturally inclined toward sustainability unless distorted by modern socioeconomic systems; on the other hand, that human nature is inherently destructive, posing a fundamental barrier to environmental action. We critically examine these presuppositions, their foundations, as well as their pro- and counterarguments, and argue that both are oversimplifications which overlook the current understanding on biological, evolutionary and behavioral sciences, disregarding its contextual nature. Many native populations have overexploited their resources, yet modern evolutionary psychology does not support the notion that human nature would be inherently unfit for environmental action. Evolved behavioral tendencies interact with socioeconomic environments which can lead to the relational properties of environmental destruction as well as to protection. Their high behavioral variability, interactivity, calibration, flexibility, plasticity, and co-optability enable a wide range of sustainable actions. Rather than seeing biological and evolutionary aspects as inherently pessimistic or optimistic *per se*, we call for more research which appropriately integrates behavioral biology and evolutionary psychology so that we can avoid the above-described erroneous presuppositions as well as related Moralistic and Naturalistic Fallacies. We also argue toward a more nuanced understanding of human nature, and thus design more effective interventions which fit our biological predispositions. Furthermore, promoting education, ethical control and responsible journalism may help to avoid fostering these misguided assumptions about human nature. We conclude that evolved, universal psychological tendencies neither justify inaction nor make sustainability unattainable. Instead, correctly understanding human nature serves as a crucial foundation for guiding us toward designing effective and lasting sustainable practices.

## 1 Introduction

Humanity is currently facing intersecting political, economic, social and environmental crises. In the environmental system, the most important threats are natural resource depletion, biodiversity loss, pollution, and global warming. The convergence of these interconnected threats has led to a worldwide polycrisis, posing potentially devastating risks for the Earth and its many inhabitants species (Lawrence et al., 2024). This polycrisis is driven by various anthropogenic factors, including, but not limited to: (1) the unsustainable extracting, consuming, and wasting of natural resources and land, particularly exacerbated by hyper-consumerism in affluent societies, leading to the depletion and degradation of both terrestrial and aquatic ecosystems (Abbass et al., 2022; Richardson et al., 2023; Ripple et al., 2023, 2024); (2) the release of toxic substances from industrial, agricultural, urban and domestic activities, such as plastic waste, pesticides, and persistent chemicals, at levels exceeding the planet’s natural capacity to absorb and neutralize them (Persson et al., 2022; Richardson et al., 2023); and (3) the emissions of greenhouse gasses from fossil fuels, biomass combustion, livestock, landfills, fertilizers, refrigerants, and wildfires (IPCC, 2023; Ripple et al., 2023, 2024). Our population size, globalized market, contemporary societal institutions, inequality, warfare, overexploitation, and cognitive biases are among the cultural and psychobiological drivers that contribute to ecoclimate problems (King and Jones, 2025; Rees, 2008).

Consequently, six of the nine crucial planetary limits that support and regulate life on Earth (viz. biogeochemical flows, freshwater change, land system change, biosphere integrity, novel entities, and climate change) have already been breached (Richardson et al., 2023). Some researchers suggest that we have initiated the Sixth Mass Extinction (Ceballos et al., 2017; Naggs, 2017), and that there is a significant likelihood of forest ecosystem collapse within the next 40 years (Bologna and Aquino, 2020). We are already experiencing an increased frequency of record-breaking heat-waves, extreme weather events, rising sea levels, and ocean acidification, all of which pose severe threats to both ecological and human systems (IPCC, 2023; Ripple et al., 2023, 2024). Low-income populations are disproportionately affected, exacerbating inequalities (Guedes et al., 2024), increasing outbreaks of tropical diseases, such as dengue, malaria and cholera (Kaseya et al., 2024), and increasing climate-induced migration flows worldwide (Abbass et al., 2022; Guedes et al., 2024). Furthermore, land-use changes and pollution weaken ecosystems’ ability to provide essential services that sustain a broad range of human needs (Fuller et al., 2022; Persson et al., 2022; Ripple et al., 2022, 2023, 2024), adversely impacting all facets of ecosystems with profound implications for both biome integrity and public health (Agache et al., 2022; Ripple et al., 2022, 2023, 2024).

In principle, we know many pathways to mitigate and solve environmental crises: lowering the rates of extraction and consumption of natural resources, de-intensifying land use, curbing pollution, restoring ecosystems (Griscom et al., 2017; Silva et al., 2023b; Tavares et al., 2024), and lowering atmospheric carbon concentrations (Van der Gaast and Begg, 2012; IPCC, 2023). These goals can be achieved through various means, from market regulation, international agreement, treaties, and cooperation (Silva et al., 2019) to transitioning toward circular economies, tackling planned obsolescence and consumerism, and adopting sustainable, efficient waste management practices (Van der Gaast and Begg, 2012; IPCC, 2023). We are already witnessing many impressive initiatives in action, such as the EU Green Deal, the “grain for green” program in China (Ma et al., 2024), regeneration of tropical natural vegetation in the Brazilian Atlantic Forest (Silva et al., 2016; Silva et al., 2023a), and diverse technological advances and dramatic cost reductions in renewable energy (Tawalbeh et al., 2021; Breyer et al., 2022). Some evidence even suggests a certain degree of decoupling between economic growth and greenhouse emissions (Wang et al., 2018; Wu et al., 2019; Freire-González et al., 2024; Infante-Amate et al., 2024). Yet, it is clear that these efforts fall short of what is needed to halt and reverse the ongoing environmental crises. Neither current technological advances, governmental policies, nor international agreements are sufficient to achieve net zero by 2050 (Marteau et al., 2021), and the decoupling of economic growth and greenhouse emissions remains limited (Freire-González et al., 2024) while there seems to be no evidence of decoupling between economic growth and the use of natural resources (Parrique et al., 2019).

The need to understand and tackle the failures to adequately address environmental polycrisis calls for the mobilization of all research fields, including the behavioral sciences (Steg, 2023). Psychology has already established itself as a crucial discipline for promoting human well-being, namely, through contributions in health and education by promoting healthy behaviors and improving teaching methods. Moreover, its potential to offer evidence-based interventions makes psychology well-positioned to play a central role in developing strategies that foster changes toward sustainability (Steg, 2023). Ultimately, environmental (and non-environmental) behaviors hinge on the actions of individuals (Hampton and Whitmarsh, 2023), be them consumers, workers, capital owners, investors or policy-makers, putting psychology and behavioral sciences in a pivotal role in fostering required behavioral shifts and identifying what structural changes are needed to bring about these shifts (Hoffman, 2010; Marteau et al., 2021; Nielsen et al., 2021; Whitmarsh et al., 2021; Steg, 2023).

Psychology and the behavioral sciences have already made significant strides in understanding and promoting pro-environmental behaviors, as well as in enhancing the ability to adapt and recover from environmental catastrophe, focusing on the human dimensions of the environmental issues such as climate change (Whitmarsh et al., 2021; Marteau et al., 2021; Nielsen et al., 2021, 2024; Steg, 2023). On this topic, there are already book-length contributions (Gardner and Stern, 2002; Koger and Winter, 2011; Clayton and Manning, 2018; Amel et al., 2021; Gatersleben and Murtagh, 2023), systematic reviews (e.g., Boluda-Verdu et al., 2022; Flores et al., 2024; Tam et al., 2021), meta-analyses (e.g., Morren and Grinstein, 2016; Nisa et al., 2019; Soutter et al., 2020; Cipriani et al., 2024), and even a second-order meta-analysis (Bergquist et al., 2023). The literature on the psychology of the climate crisis identifies a range of predictors for pro-environmental attitudes and behaviors, including personality traits such as honesty-humility, openness, agreeableness, conscientiousness, and extraversion (Soutter et al., 2020; Cipriani et al., 2024), liberal political ideology (Cruz, 2017), connection to nature (Whitburn et al., 2020), and heightened perceptions of climate change risk (Lacroix and Gifford, 2018).

Yet, misconceptions about human nature abound in disparate literatures, hindering the progress of the behavioral sciences in general (Hagen, 2005; Sosis, 2009; Varella et al., 2013; Jonason and Dane, 2014; Buss and Von Hippel, 2018). In this article we argue that the progress of psychology and behavioral sciences in solving environmental issues could greatly benefit from a more sober and nuanced discussion around *two pervasive misguided assumptions* about human nature, presuppositions underlying the lines of reasoning that still appear repeatedly in both scientific literature and public discourse about the environmental and climate crisis:

1. Human nature, conceived as a set of inherent noble savage-like traits, is ultimately environmentally friendly and, therefore, ideal for effective environmental action when not exposed to destructive modern socio-cultural environments.
2. Human nature, understood as a collection of universal problematic psychological and behavioral traits, is ultimately environmentally destructive and, as such, constitutes an inherent barrier to effective environmental action.

In this article, we critically examine these *two pervasive misguided assumptions*, their origins, current expressions as well as supporting and opposing arguments. Rather than being a perfect black or white dichotomy, these two erroneous presuppositions form a continuum, and most academic views explicitly or implicitly fall between these two extremes rather than embracing either position fully. Yet examining the polar cases proves analytically valuable. By deconstructing the extreme endpoints, we fundamentally challenge the validity of the entire misguided conceptual continuum instead of merely building straw-man cases to promote a golden middle ground. Through conceptual analysis and review, we demonstrate how both opposing presuppositions stem from mischaracterization of behavioral phenomena disregarding their contextual nature, resulting in either an overly pessimistic or an overly optimistic outlook on the prospects for achieving sustainability. Since addressing environmental challenges requires an accurate understanding of human nature within a well-informed biocultural approach, it is crucial to move toward a more nuanced, up-to-date debate, one that incorporates modern evolutionary psychology as well as interdisciplinary and integrative perspectives. In this biocultural approach, human behavior is acknowledged as a product of both biological and cultural influences, which interact in specific ways and may produce both environmentally beneficial and environmentally destructive outcomes in relation to local conditions.

Hence, this article aims to clarify and advance the debate on human nature in the context of environmental and climate crises, informing researchers across disciplines about prevailing misconceptions, and contribute to the interdisciplinary discussion on development of efficient pro-environmental interventions that are aligned with and tailored to the human mind. Here, we do not argue that understanding human nature without both misconceived presuppositions will alone promote pro-environmental action and solve ecoclimate problems, but rather that by promoting a more informed and nuanced discussion about the actual role of human nature we might avoid unnecessary controversy in the literature due to conceptual polarization and aid future research promoting the development of more effective interventions to the polycrisis. We acknowledge that psychological approaches, although crucial, represent just one dimension of addressing the polycrisis, which equally demands technological, economic, political, diplomatic and institutional solutions (Van der Gaast and Begg, 2012; IPCC, 2023). By clarifying these parameters, we aim to steer discussions toward the future production of more nuanced and effective intervention strategies.

## 2 The concept and evidence of the ecologically noble savage

Rousseau (1755) depicted the natural state of humans as inherently good, virtuous, and free, living harmoniously in a state of environment prior to the corruption introduced by non-tribal society. This “noble savage” concept has been widely adapted and explored, particularly within modern environmentalism (cf. Redford, 1991; Ridley, 1997; Krech, 1999; Van Vugt et al., 2014), often with the conclusion that Indigenous peoples are inherently inclined to live in harmony with nature and preserve ecosystems (Alvard, 1993; Hames, 2007a). This view, which portrays Native Americans as original conservationists, gained prominence through early conservationist writings and was later embraced by cultural ecology theories (Redford, 1991; Alvard, 1993; Krech, 1999; Hames, 2007a).

The ecologically noble savage repackages the old idealized notion of “noble savage” (cf. Keeley, 1997, 2014) with outdated ecological notions, such as living “in balance” or “in harmony” with nature, to promote the conclusion that native peoples living in traditional lifestyles, free from Western influence, “respect nature” and live “in close harmony” with their environment (Redford, 1991; Krech, 1999). For instance, “Indigenous people demonstrate a concern for maintaining the ecological processes and the species that mediate those processes. (…) The commitment of indigenous peoples to conservation is complex and very old” (Alcorn, 1993, p. 425). Another more recent appearance of the ecologically noble savage can be found in the literature against eco-colonialism. For example, “indigenous cultural epistemologies (…) encompass indigenous knowledge systems, traditional practices and performances, and cosmologies that embody sustainable ways of living and harmonious relationships with the natural environment” (Ohenhen and Abakporo, 2024, p. 2). The authors argue that the “traditional ecological wisdom” conveyed by the indigenous elders “encourages the community to live in harmony with the environment and fosters a mentality shift toward eco-conscious living by acting as a guide for sustainable activities” (Ohenhen and Abakporo, 2024, p. 12). Moreover, the authors state that “the Cree people of North America (…) emphasizes the need to preserve harmony and balance with nature as well as the interdependence of all living things” (Ohenhen and Abakporo, 2024, p. 12). Although well-intentioned in highlighting alternative ecological paradigms, these narratives romanticize in different degrees Indigenous relationships with nature by promoting an uncritical, intrinsically-sourced and homogenized view of Indigenous sustainable ecological engagement. It also neglects historical evidence of resource depletion and obscures the dynamic, context-dependent ways in which human societies, Indigenous and non-Indigenous alike, interact with ecosystems (Smith and Wishnie, 2000).

Some examples seem to support the notion of Indigenous and traditional peoples avoiding overexploiting their environment. Hunter-gatherers in the Pacific Northwest exercise forms of hunting control (Smith and Wishnie, 2000), and Pacific Islanders designed marine conservation techniques long before Western societies (Johannes, 1978, 2002b). Indigenous societies in the Amazon avoid deforesting areas near riverbanks (Smith and Wishnie, 2000) and manage their environment in ways that create large areas dominated by useful species or increasing regional tree diversity (Balée, 1993; Clement et al., 2015; Levis et al., 2017). Likewise, Aboriginal fire management practices promote habitat heterogeneity, enhancing local biodiversity by supporting ecologically keystone species critical to ecosystem functioning (Bird et al., 2018). At least 36% of the remaining preserved forests worldwide are inside Indigenous People’s territories (Garnett et al., 2018; Fa et al., 2020; Fernández-Llamazares et al., 2024), and there is a strong spatial correlation between the diversity of Indigenous languages and biological diversity (Mulder and Coppolillo, 2005). Despite facing challenges such as armed conflict and land dispossession, indigenous peoples maintain strong territorial connections in several biodiversity hotspots promoting landscapes with higher ecological integrity and reduced anthropogenic impact (Beattie et al., 2023). Native and traditional peoples across the globe often exhibit a deep connection to and extensive knowledge of their territories, while maintaining a smaller overall carbon footprint compared to populations in urbanized and industrialized societies. Furthermore, by resisting the occupation of their land and becoming vocal climate change activists in national and international contexts, they play a crucial role in conservation and the promotion of sustainability (Etchart, 2017; Estrada et al., 2022). Additionally, their ecological knowledge, culturally accumulated over many generations, can contribute to, inform, and improve conservation research (Johannes et al., 2000; Albuquerque et al., 2021; da Silva et al., 2024).

Nevertheless, evidence suggests that Indigenous and traditional peoples can also overexploit their environments to different degrees, prioritizing the short-term benefits of natural resources extraction over long-term ecological considerations (Smith and Wishnie, 2000; Hames, 2007a,b). For example, Alvard (1993) describes how the Piro people, a group of subsistence hunters of Amazonian Peru, harvest species that are vulnerable to over-hunting and local extinction, making decisions that align with optimal foraging theory, specifically by maximizing the short-term harvesting rate rather than long-term sustainability. Similarly, Hames (2007b) reviews a series of empirical studies showing that Native Amazonians place significant value on protein, driving them to intensify their hunting efforts to obtain it or extend their hunting ranges even amidst resource depletion. Indeed, the exploitative potential of Native populations can be tracked back in history as far as to the Late Pleistocene global human expansion and the era of island colonization (Boivin et al., 2016; Araujo et al., 2017; Prates and Perez, 2021), characterized by human-induced population crashes and extinctions of large mammals in particular (Turney et al., 2008; Bartlett et al., 2016; Bergman et al., 2023; Lemoine et al., 2023). Overexploitation of environmental resources also likely played a significant role in the collapse of many ancient societies (Janssen and Scheffer, 2004; Diamond, 2011). Humans are great niche constructors (Albuquerque et al., 2018) who exhibit a sustained pattern of habitat modification, hunting, and intensive harvesting, particularly of terrestrial vertebrates and marine invertebrates, for at least the past 50 ka years (Sullivan et al., 2017). Even earlier, around 1.8 Ma (i.e., Megaanni; million years) ago, hominins accelerated the extinction rates of several elephant-like species by a factor of five (Hauffe et al., 2024). Thus, it appears clear that human nature, even when kept free of modern socio-cultural structures, rarely gravitates toward voluntary resource conservation as a “prudent” forager regardless of the material context (Smith and Wishnie, 2000).

## 3 The concept and evidence of a biologically doomed species

In contrast to philosophers like Rousseau and Kropotkin, who viewed human nature as fundamentally virtuous and altruistic, thinkers such as Hobbes and Machiavelli argued that human nature is inherently selfish and individualistic (Ridley, 1997). The inclusion of Malthus, Huxley and Skinner in the latter group further reinforced the link between selfishness and psychobiological determinism (Ridley, 1997; Gardner and Stern, 2002). Williams (1966) critically dismantled the then-dominant notion of animals acting selfless “for the good of the species,” challenging this naive form of group selectionism. Dawkins (1976) later painted a convincing picture of the sociobiological revolution as a dynamic interplay of altruistic and selfish behavioral patterns, all ultimately rooted in the evolutionary logic of selfish genes. Despite what the title might suggest at first sight, The Selfish Gene (Dawkins, 1976) is about the evolved reality of altruistic tendencies, and despite defining selfishness in a technical behavioristic way, some readers misunderstood it as meaning emotionally, motivationally or that the nature of humans is exclusively self-interested, advocating an egoistic view of ethics (Dawkins, 1981).

In fact, evolutionary pressures shape the behavioral predispositions of all species toward increased inclusive fitness (Alcock, 2001; Ågren, 2021). As a result, humans, like all animal species, could be expected to exhibit foraging strategies and psychological traits focused on short-term survival and efficient resource acquisition (Alvard, 2007; Hames, 2007b; Schaller et al., 2017), ultimately linked to the maximization of reproductive success of individuals, their kin and reciprocating allies (Williams, 1966; Alcock, 2001; Trivers, 2006; Ågren, 2021; see also Bates and Lees, 1979). In this sense, there have been many advances in explaining the evolution of human altruistic inclinations including mechanisms such as kin selection, direct reciprocal altruism, indirect reciprocity, signaling theory (Bliege Bird and Smith, 2005; Kurzban et al., 2015; Henrich and Muthukrishna, 2021). Conversely, humans are a highly social species (Richerson and Boyd, 1998; Wilson, 2012) and have evolved many propensities toward social interdependence (Migliano and Vinicius, 2022; Syme and Balliet, 2025). While foraging, humans can even cooperate with wild species such as birds, dolphins, wolves and orcas (Cram et al., 2022), and there is convergent evidence from experiments, surveys, field observations, and anthropological studies that challenge the “Homo economicus” notion, which posits that humans are only motivated egotistically without any genuine or disinterested prosocial tendencies (Hill et al., 2009; Arbuthnott, 2025). Furthermore, the contextual factors that tend to lead individuals toward less cooperation and more selfishness, such as wealth, power, distrust, and fear of being exploited, are known to psychological sciences and should be taken into account when designing pro-environmental interventions (Arbuthnott, 2025). At the same time, the cooperative nature of humans and the factors leading to selfishness are not widely known which might contribute to delaying sustainability goals (Arbuthnott, 2025).

The theoretical frameworks of classical sociobiology and rational choice economics, particularly through optimization models and game theory, might have helped foster a pessimistic outlook on human capacity to manage shared resources (Mulder, 1988; Smith et al., 2001; Hill et al., 2009). In that sense, the “tragedy of the commons” refers to the dilemma between individual and collective interests: when individuals act in their immediate self-interest by overexploiting a common-pool resource, anticipating that others will do the same, the collective outcome is resource depletion. Well-documented historical and contemporary cases validate this pattern in different regions (Ridley, 1997; Gardner and Stern, 2002). For Hardin (1968), “freedom in a commons brings ruin to all” (p. 1244). He mentioned overgrazing, overfishing and over-pollution as inevitable outcomes of the tragedy of commons aligned with over-population and he argued that this problem admits no purely technical solution, requiring instead a radical expansion of human morality. For decades, many have interpreted this analysis as “proof” that humans are biologically destined to degrade their environments, a conclusion that conflates game-theoretic predictions among independent players with presumed real-life ecological inevitability.

However, the tragedy of the commons can be mitigated through institutional and group arrangements facilitating open communication about collective needs and creating self-regulation via a penalty system to limit over-extraction before the tragedy of commons becomes dire reality. Specifically, experimental evidence regarding shared ground water, forest and ocean resources demonstrates that structured dialog enables resource users to establish trust, develop cooperative norms, and implement monitoring systems against free-riders that align individual incentives with sustainable outcomes. Case studies of successfully managed commons reveal that such systems emerge most effectively through spontaneous participatory, community-based self-governance rather than externally enforced regulation (Ostrom, 1990, 2000; Gardner and Stern, 2002; see also Hardin, 2007). For instance, the Turkana pastoralists of Kenya exhibit social institutions and management practices that do not degrade their local environment (McCabe, 1990; but see Ruttan and Mulder, 1999). This demonstrates how social frameworks can transform Prisoner’s Dilemma scenarios into cooperative games without requiring fundamental moral transformation. The findings underscore that environmental sustainability depends not on inherent human traits but on situational factors enabling collective action. In 2009, Elinor Ostrom was the first woman to win the Nobel Prize in Economic Sciences for her analysis of economic governance of the commons, showing that the tragedy of the Commons is not inevitable. However, her advances are still not widely known.

Still, technically speaking, humans have not evolved “to” preserve the environment (Hames, 2007a,b; Mulder and Ruttan, 2007). But this does not mean that we are incapable of doing so, the same way we can ride a bicycle despite not “evolving to” do so. Nevertheless, this poorly understood biological perspective has given rise to the concept that is a polar opposite of the “ecologically noble savage” notion, namely that human nature is inherently selfish and destructive to the environment and as an obstacle to environmental action, seemingly leading to the conclusion that unsustainability is an unavoidable and justifiable outcome (Gardner and Stern, 2002; Atkinson and Jacquet, 2022). The same narrative has also been observed in various forms in individuals’ narratives on inaction, for instance, in the form of “societal change is not possible because most people are too selfish and lazy to act” (Cherry et al., 2024).

As documented by Atkinson and Jacquet (2022), this claim that humans are not naturally designed to solve climate change frequently appears in public discourse, journalism and academia, under article titles such as “The dragons of inaction: Psychological barriers that limit climate change mitigation and adaptation,” “What if We Stopped Pretending the Climate Apocalypse Can Be Stopped?,” “Study Shows That Human Beings Are Too Selfish to Fix Climate Change” or “Do not even think about it: Why our brains are wired to ignore climate change” (Gifford, 2011; Marshall, 2015; Atkinson and Jacquet, 2022). However, by cherry-picking the quotations Atkinson and Jacquet (2022) are forcing the conclusion that whenever an author says that humans did not evolve to solve climate change they necessarily mean that it is impossible to do so, as a justification for inaction. Furthermore, they generalize this forced conclusion across journalistic sensationalism and articles of expert academics, including evolutionary psychologists. Yet, evolutionary psychologists and those psychologists who understand evolution clearly distinguish between what humans evolved to do and what humans are actually capable of doing. For instance, Gifford (2011) lists seven general psychological barriers to climate change mitigation and adaptation and 29 specific manifestations of those barriers. Among the manifestations of “limited cognition,” he includes “ancient brain” because “our ancestors were mainly concerned with their immediate band, immediate dangers, exploitable resources, and the present time” (p. 291). This is a simplistic view of our ancestors underestimating their capacity for future planning, cooperation and self-regulation. However, he readily admits that this does not imply impossibility: “Obviously, our ancient brain is *capable* of dealing with global climate change, but doing so does not come easily” (Gifford, 2011, p. 291). Likewise, Wilson et al. (2007) agree that “(…) *Homo sapiens* is not by nature a conservationist (…)” (p. 32), however they also acknowledge that the “suggestion that “human nature” is a source of environmental exploitation and degradation is not a claim that nothing can be done (…) (p. 51). Similarly, Van Vugt et al. (2014) claimed that our minds “are not designed to respond to environmental problems (…)” (p. 23–24), but before that they have said that an “evolutionary perspective does not imply genetic determinism” (p. 7). By the same token, Victor et al. (2017) stated that our brains “are unfortunately not wired to tackle problems like climate change. With some help we can build policies that enable us to do better.” Atkinson and Jacquet (2022) did not mention those caveats and nuanced perspectives from each of the mentioned authors painting an unfair and biased picture of experts in evolutionary psychology. Even Ridley and Low (1993) were misquoted by Atkinson and Jacquet (2022) as if they were normalizing climate inaction as inevitable, but in fact Ridley and Low (1993) followed that same nuanced route by arguing that “(…) human beings are motivated by self-interest rather than collective interests. But that does not mean that the collective interest is unobtainable (…)” (p. 4). Interestingly, Atkinson and Jacquet (2022) themselves also follow this nuanced line of reasoning by arguing that “Of course, there is a sense in which humans did not evolve to solve climate change, just as we did not evolve to read, sit at desks all day, live in cities, scuba dive, or have gender equality. Culture allowed for these behaviors. At the same time, we are not *not* evolved to deal with climate change. The psychological features that have made us uniquely able to cause this problem also make us uniquely capable of solving it” (p. 8). By overlooking these nuanced positions, such critiques miss the fundamental biocultural reality that evolved psychological capacities shape but do not impede or fully determine human responses to climate change, as our species’ distinctive capacities for cultural innovation and institutional evolution provide multiple pathways to sustainable futures when properly mobilized.

## 4 What do modern evolutionary, biological, and behavioral sciences say?

Both of the misguided assumptions described above contradict core principles of biological and evolutionary science. They also clash with the tenets of evolutionary psychology and other behavioral sciences. Modern biology is not deterministic in the sense as it is often portrayed, given that it recognizes the complex interplay between genotype and environment in shaping phenotypes, along with the role of randomness and multiple simultaneous, interacting causal factors (Ridley, 1997; Mayr, 2004; Harden, 2023). These factors include alternative splicing,^1^ epigenetics,^2^ incomplete penetrance,^3^ and variable expressivity,^4^ each emphasizing variability, interactivity, and plasticity.^5^ This inherently probabilistic framework suggests predispositions, susceptibilities, and propensities rather than rigidly determined or “unavoidable” outcomes (Ridley, 1997; Mayr, 2004; Valentova and Varella, 2016). As behavioral plasticity is often favored by natural selection in long-lived species, we have evolved psychological mechanisms which are calibrated to local environmental conditions throughout ontogenesis,^6^ leading to acclimatization,^7^ and producing a wide range of behavioral outcomes based on individuals’ unique experiences, sociocultural contexts, and other situational factors (Hagen, 2005; Confer et al., 2010; Brown et al., 2011; Nettle et al., 2013; Tooby and Cosmides, 2016). In other words, the biological, evolutionary and psychological predispositions underlying human behavior are inherently interactive and flexible, which can lead to either environmental conservation or destruction in a relational manner, depending on the prevailing local conditions.

In this sense, it is crucial to recognize that both the romantic view of humans as inherently “ecologically noble” and the cynical view of humans as biologically destined to exploit nature commit the same cognitive error: the correspondence bias or the fundamental attribution error, which is the pervasive tendency to overattribute the causes of behavior to dispositional traits while underestimating the contributions of the situational context (Gilbert and Malone, 1995). This “dispositionism” seems to be an evolved universal mode of thinking (Choi et al., 1999; Andrews, 2001). Each misguided assumption of human nature mistakenly treats sustainability or destructiveness as internal dispositional traits, when in reality they are emergent relational outcomes shaped by situational context (see Rees, 2008). Human nature comprises both cooperative and exploitative tendencies (Pinker, 2012; Sapolsky, 2017), but neither a selfless ecological nobility nor irredeemable selfish ecological destructiveness, rather adaptations that respond to environmental and social conditions (Hagen, 2005; Confer et al., 2010; Brown et al., 2011; Nettle et al., 2013; Tooby and Cosmides, 2016). These opposing preconceptions ignore the core insight of evolutionary behavioral science, that humans evolved the tendency of expressing context-dependent strategies. By overemphasizing dispositional explanations while neglecting situational factors, both narratives erroneously convert dynamic, emergent relational properties, such as sustainable or exploitative behaviors, into stable, inherent traits challenging the validity of the entire misguided conceptual continuum. We argue that no position that views human nature as in any degree inherently inclined to ecological sustainability or overexploitation is factually correct given that those are emergent relational outcomes to particular constellations of socio-ecological situational factors with which human nature interacts. Thus, the entire conceptual continuum from inherent ecologically noble savage to inherent ecological overexploitation is misguided. Recognizing that it is mistaken to view sustainability or destructiveness as dispositions within human nature helps avoid the pitfalls of essentialism, determinism, and fatalism allowing for more productive approaches to fostering pro-environmental behavior through designed contexts rather than appeals to presumed inherent tendencies.

Moreover, evolutionary biology and psychology explicitly rejects essentialism, defined as the belief in fixed, internal essences for groups or categories (Mayr, 2004; Dennett, 2017; Varella et al., 2013; Varella, 2016; Tooby and Cosmides, 2016), allowing for a dynamic understanding of human nature (Hagen, 2005; Confer et al., 2010; Brown et al., 2011; Nettle et al., 2013). Instead, evolutionary psychologists and biologists place greater explanatory value on populational thinking^8^ and on evolved regulatory/operational mechanisms (genes, epigenetics, physiological, and psychological) that produce adaptive behavior in response to the environment. Thus, the often-cited psychological “barriers” obstructing sustainable behaviors should not be viewed as impeditive barriers in the sense of insurmountable obstacles independent of the sociocultural context (Schmitt et al., 2020; but see Atkinson and Jacquet, 2022), but rather as barriers in the sense of relational challenges between human nature and the sociocultural context, requiring attention to the sociocultural conditions at hand. The assumption of “unavoidable” environmental destruction presupposes a degree of determinism akin to that found in Laplacean physics, which is not supported by evidence in either psychology or biology (Ridley, 1997; Pinker, 2003; Mayr, 2004). As Hagen (2005) explains, the existence of a determined biological mechanism, such as the skeleton, does not equate to a fixed and predetermined behavioral output of this mechanism. A useful analogy is human behavior and computer programs that play virtual chess: even though the chess rules are fixed, and programs have many predetermined “if-then” logics, each specific chess match is unique because, while following fixed rules, interacting players open and close many courses of action throughout the match. In a similar way, the human mind comprises dispositional cognitive mechanisms following universal “if-then” logic that can generate a variety of outputs influencing the probability of exhibiting behaviors and outcomes depending on ontogenetic and situational contexts (Confer et al., 2010; Tooby and Cosmides, 2016; Pietraszewski and Wertz, 2022). Furthermore, it is crucial to note that, as humans are a highly social species, the selective pressures toward foraging strategies that maximize inclusive fitness do not imply that selection would always favor intensified foraging practices by individuals alone. In the hunter-gatherer groups of our evolutionary history, it was crucial for an individual’s fitness to retain the support and social acceptance of the group, and as a result we have evolved biological predispositions toward cooperation and morality as well as to respond to social pressure (Ridley, 1997; Ostrom, 2000; Johannes, 2002a; Hill et al., 2009; Curry et al., 2019; see also Alvard and Nolin, 2024). One could thus also argue, based on biological and evolutionary arguments, that we are ultrasocial (Richerson and Boyd, 1998; Wilson, 2012), thus also inherently inclined to be steered by local ethics, taboos, and other types of social control, including traditional conservation or environmental ethics (Ostrom, 2000; Johannes, 2002a; Hill et al., 2009; Curry et al., 2019).

Indigenous ecological wisdom often promotes ideals of sustainability and reverence for nature (Redford, 1991; Krech, 1999; Alvard, 1993; Hames, 2007a; Ohenhen and Abakporo, 2024), but this does not mean that individual foraging behavior always aligns with these principles (Low, 1996), given that studies show that foragers frequently prioritize short-term gains when opportunities arise (Alvard, 1993, 2007; Hames, 2007a,b), as all humans do. However, this individual opportunism does not render their traditional cultural values hypocritical, nor does it inevitably lead to collective environmental degradation. As shown above, it cannot be denied that Native and traditional populations have often succeeded in ways that modern populations frequently failed, namely, in being collectively less detrimental to their local environments (Levis et al., 2017; Albuquerque et al., 2021; da Silva et al., 2024). The crucial question is: If there is no underlying evolutionary drive toward sustainability or conservation, where do their apparent successes originate? One possible answer is that what is frequently interpreted as a product of an inherent conservation ethic and voluntary self-retrainment among Indigenous and traditional peoples instead emerges primarily from material constraints and extrinsic social, demographic, and cultural factors, such as low population densities, semi-nomadic lifestyles, limited extraction technologies, and environments with resources that are difficult to deplete and pollute that keep exploitation within ecological limits, rather than from intentional individual prudent foraging (Alvard, 1993, 2007; Low, 1996; Smith and Wishnie, 2000; Hames, 2007a,b). Indeed, Low’s (1996) cross-cultural analysis of 186 societies revealed that ecological constraints, rather than cultural attitudes or sacred prohibitions are the primary determinants of resource extraction practices, and that the characteristically low environmental impact of many traditional societies stems from extrinsic limiting factors (e.g., low population density, limited extraction technologies, and absent market incentives) not exceeding the carrying capacity of their local environment. Consequently, while nature-revering narratives are culturally widespread, their presence shows no significant association with actual ecological outcomes across cultures. In general, ecological variables, such as the exploration potential and the exploitation difficulty of a given natural resource, are key factors that control the social behavior of resource exploiters, both human and non-human predators (Monk et al., 2018). Another possible answer is that, in terms of cultural evolution, societies may have experienced or foreseen resource constraints or collapses, environmental catastrophes and, in response, developed forms of conservation ethics and practices to limit resource use (Johannes, 2002a). Moreover, biophilia, which is our evolved affinity for nature that leads to humanity’s widespread esthetic and emotional responses to natural landscapes, might partially explain why some small-scale societies develop cultural traditions and practices that foster reverence to nature and sustainability, as their daily lives remain intimately tied to ecosystems they instinctively value (Gardner and Stern, 2002; Wilson, 2007; Barbiero and Berto, 2021). While indigenous groups may articulate profound respect for nature and achieve sustainable collective outcomes, this might reflect situational and practical circumstances more than individual restraint, challenging the romanticized notion of an inherent virtuous and sustainable human nature within the “ecologically noble savage” without dismissing the conservation values of their traditional knowledge and their collective sustainable outcomes. At the same time, further evidence is needed to determine the extent to which different extrinsic factors contribute to sustainability of resource use by diverse Native and traditional populations across the globe. Yet, one thing is certain: Even if, as evidence suggests, humans lack an inherent evolutionary biological and/or psychological drive toward conservation or sustainability, we certainly have the potential, under favorable socio-ecological conditions, to exhibit pro-environmental behaviors.

Accusations of essentialism often stem from intuitions from folk biology and folk psychology, and are rooted in psychological essentialism, an intuitive mode of categorical thinking that interferes with the accurate understanding of biology, evolution, and genetics (Gelman and Rhodes, 2012; Dennett, 2017; Dar-Nimrod et al., 2021). Psychological essentialism reinforces misconceptions by fostering beliefs in the stability of categorical distinctions, intensifying perceived boundaries, assuming within-category homogeneity, attributing inherent causal properties to individuals, and endorsing idealized category prototypes (Gelman and Rhodes, 2012). This essentialist cognitive bias is readily activated when individuals naïvely engage with biological topics or concepts, often hindering the comprehension of evolutionary principles and perpetuate stereotypes and prejudices (Gelman and Rhodes, 2012; Neufeld, 2022). Notably, research indicates that greater knowledge of modern genetics reduces essentialist tendencies and can even mitigate the influence of sensationalist headlines about genetics (Dar-Nimrod et al., 2021). Thus, evolutionary psychology and modern biology together can promote a nuanced, non-essentialist understanding of human behavior and diversity, grounded in scientific evidence.

Rather than being supported by scientific evidence, the misconceptions of inherently destructive or inherently ecological human nature persists due to the misuse and oversimplification of biological and evolutionary facts, often influenced by folk biology and folk psychology. These cognitive tendencies are frequently employed to support political and/or ideological views on how we should (or should not) react to environmental destruction, thereby perpetuating stereotypes and prejudice (Gelman and Rhodes, 2012; Neufeld, 2022; see also Confer et al., 2010; Hagen, 2005). Particularly in public discourse, a common error is the conflation of explanation with justification, an instance of the Naturalistic Fallacy, which leads to the mistaken conclusion that what currently exists, ought to exist (Hagen, 2005; Varella et al., 2013; Birgül, 2024). Atkinson and Jacquet (2022) illustrate this issue, including media examples, suggesting that sometimes framing human nature as an inherent barrier to pro-environmental behavior gives the impression to be a rhetorical tool to justify inaction, fostering fatalism and pessimism while undermining collective efforts to address environmental challenges. Some might try to justify resource overexploitation by simplistically invoking our “selfish nature,” overlooking our evolved tendencies toward altruism, prosociality, group decision-making, environmental niche construction and ethics. Nevertheless, the human mind relies on finite cognitive mechanisms shaped by lifetime calibration and sociocultural contexts which permits vast behavioral flexibility, rejecting fatalistic pessimism.

Descriptive behavioral sciences can be seen as being, in principle, free of ethical prescriptions, normative judgments, or agendas aimed at supporting the status quo (Pinker, 2003; Varella et al., 2013; Horowitz et al., 2014; Confer et al., 2010; Buss and Von Hippel, 2018; Tooby, 2020; Buss, 2020, 2025). The misuse and misappropriation of these sciences does not render them useless or inaccurate per se; however, it remains crucial to assess whether knowledge of underlying biological predispositions may contribute to fatalistic attitudes and environmental inaction. Interestingly, it has been observed that evolutionary researchers tend to be significantly less politically conservative than the average U. S. citizen and are equally politically progressive as non-evolutionary researchers (Tybur et al., 2007). Similar findings have been reported among evolutionary anthropologists, who neither express nor promote reactionary or conservative political agendas and hold political beliefs that are highly liberal and indistinguishable from those of non-evolutionary anthropologists (Lyle and Smith, 2012). Taken together, these studies reject the idea that evolutionarily oriented researchers are actively pushing reactionary political agendas into their science given that they tend to be as liberal as non-evolutionists. In this context, the misconception of inherently destructive human nature may often serve as a *post hoc* rationalization for preexisting reactionary ideological and political views rather than genuinely steaming from evolutionary psychological sciences.

The idea of an inherently destructive human nature is amplified by what we call “Linear Transference Fallacy,” which includes and extends upon the “Fallacy of Composition” (Finocchiaro, 2015). The “Linear Transference Fallacy” is a bias where individuals subconsciously and erroneously attribute the same properties or qualities of one composite entity or collective system to its related parts or components (cf. Finocchiaro, 2015). This fallacy also occurs when people assume a direct and unchanging correspondence between causes, outcomes, or even contexts, without considering the complexity of the system as a whole. This general linear transference of qualities arises from an oversimplified intuitive mirroring process that is focused on similarities or analogies and neglects the complex transformations, interactions, degrees of freedom, and/or emergent properties that occur between levels or facets of a complex system. For instance, in the “selfish gene, selfish person” (Hagen, 2005; Varella et al., 2013), the metaphor of a “selfish gene” is often misinterpreted as implying overwhelming selfishness in human behavior, overlooking that people can also be caring and unselfish as well as the emergent complexities at the psychological, group or societal level (Dawkins, 1981; Ostrom, 2000; Hagen, 2005; see also Alvard and Nolin, 2024). Similarly, the lack of evolved prudent foraging behavior in the individual level does not mean that a collectivity of those individuals in a given socio-ecological context could not present emergent collective sustainable outcomes nor traditional conservation ethics. In the same line, a deterministic biological mechanism is frequently assumed to result in stereotypical inflexible behavior, overlooking the contextual modulation, plasticity and degrees of freedom among the components inherent in such mechanisms. Another common instance of this fallacy involves concluding from the fixed rules of programmed software its supposed incapacity for diverse and adaptive outcomes, which indeed can occur including repurposing by co-optation, depending on user interactions, settings and environmental inputs. Additionally, when a problem is identified as having biological causes, this fallacy often leads to the conclusion that it can only be addressed biologically, disregarding the potential for social, cultural, or behavioral interventions. For instance, myopia is an ocular anatomical condition, but it can be mitigated socio-culturally by encouraging outdoor activities during childhood, using corrective lenses, or undergoing refractive surgery, among others (Saw et al., 2019). Lastly, this flawed reasoning is evident in arguments suggesting that if humans did not evolve specific capacities to save the planet, then “we are inherently incapable of saving it”; or that if climate inaction does not ultimately rest much in human nature per se, but in our current cultural institutions and socioeconomic structures, then “we can safely ignore role of human nature and only focus on cultural shifts.” Atkinson and Jacquet (2022) appear to endorse the latter misapprehension when stating “(…) that the most tractable barriers to tackling climate change are not found in human biology, but in human culture” (p. 7). Thus the “Linear Transference Fallacy” along with the “Fallacy of composition” reflects a pervasive misunderstanding of how properties and processes shift across different levels of organization or abstraction, which may blind individuals to critical nuances and alternative perspectives and counterintuitive possibilities. By overcoming the “Linear Transference Fallacy,” one gets much closer to understanding the complex system theory and complex thinking, which includes non-linearity, complexity, regime shift, concepts underlying the Socioecological Systems Theory required to deal with most of the environmental problems (Biggs et al., 2022).

Recognizing and addressing these fallacies is essential for fostering more accurate interpretations and for crafting nuanced, effective solutions in science, philosophy, and everyday reasoning. For instance, the “Linear Transference Fallacy” manifests clearly in environmental policy debates, as demonstrated by Ridley and Low’s (1993) analysis of sustainability strategies. Conventional wisdom assumes that global conservation requires cultivating altruistic concern for the planetary common goods, which is a linear transfer from collective outcome to individual level virtues. Yet Ridley and Low’s (1993) convincingly argue that a more sensible approach to prompt global sustainability are systems leveraging immediate self-interest by aligning short-term rewards with sustainable behavior such as payments for ecosystem services or energy cost savings, tradable fishing quotas, or carbon credits. This counterintuitive conclusion undermines the assumption that micro-level solutions must mirror macro-level motives, exemplifying how the “Linear Transference Fallacy” distorts and possibly delays pro-environment intervention design.

## 5 Discussion

In this article, we have conceptually examined two pervasive and *misguided assumptions* situated along a continuum of optimistic and pessimistic views about human nature: that it is inherently environmentally friendly, and as such, ideal for effective environmental action; and on the other hand, that it is inherently environmentally destructive and thus forms an evolved barrier to environmental action. Within our biocultural perspective, we have shown how both these concepts along the continuum of optimistic and pessimistic positions are misguided and oversimplify the current scientific understanding about human nature into a polarized debate which currently hampers open, fact-based discussion on the possible effective ways to tackle environmental crises. While our evolved psychological capacities can indeed pose challenges to environmental actions under certain socioeconomic conditions, human nature is nevertheless capable of promoting pro-environmental action given the favorable conditions aligning incentives with sustainability. That is because human nature exhibits remarkable plasticity and co-optability that allows pro-environmental norms to emerge when particular socioeconomic and cultural systems properly engage our evolved interdependence, innovative, cooperative, communicative, negotiating, and regulative capacities. Our species has the unique ability to transform social structures which in turn reshape individual actions into the desired collective outcomes, a biocultural feedback loop that makes sustainability possible. Crucially, we demonstrate that no position that views human nature as inherently inclined in any degree to sustainability or overexploitation is correct given that these relational outcomes emerge dynamically from specific sociocultural and ecological contexts that engage different aspects of our evolved capacities. This highlights the need to acknowledge the nuanced interplay between biology and culture.

### 5.1 The (im)possibility of flying as an illustration of the misguided assumptions

Some 350 years ago one might have argued that people are not made to fly; clearly our anatomical features were not designed to take off and stay in the air, so we might as well give up the whole idea. Fast forward to the present, and we see that humans have conquered the skies in remarkable ways, from paraglides and wingsuits to hot air balloons and zeppelins, not to mention helicopters, aeroplanes, and even spaceships venturing beyond the atmosphere. Although human anatomy did not evolve for flying, we have used our evolved brains and hands for creating physical contraptions, economic conditions, urban structures and cultural concepts that eventually did allow us to fly. And if one had listened to the biological pessimist and succumbed to the Naturalistic Fallacy and its fatalism, all this might have never been made possible. Similar risk that is real also for developing effective measures to save the environment.

Yet, the eventual success of flying also required avoiding another, equally risky fallacy, namely, the moralistic one. The Moralistic Fallacy occurs when someone rejects or suppresses facts because they conflict with their moral or ideological beliefs (Horowitz et al., 2014; Johnson, 2018; Ondráček, 2018), and, understandably, anyone making overly optimistic assumptions about human anatomy and aerodynamics, in the belief that humans *must* be able to fly, would have been guaranteed a crash landing. Attempts to fly eventually succeeded precisely because they acknowledged biological limitations of our species (avoiding The Moralistic Fallacy) as well as the socio-cultural possibilities that could overcome these limitations (avoiding the Naturalistic Fallacy). The modern means to airborne travel are all characterized by technical solutions which are tailored to the capacities and specifications of the human mind and body, from the design of boarding staircases and passenger seating to weight allowances, cockpit displays, and the emergency measures, and without such careful customization, achieving the scales of contemporary air travel would not have been impossible. Humans did certainly not evolve to fly, but arguing that flying is a purely cultural phenomenon would be a drastic oversimplification given that many evolved tendencies are co-opted in the correct combination to enable flight, from sense of direction to motor and social coordination. This historical example underscores a critical point: acknowledging natural limitations does not preclude overcoming them, just as denying these natural limitations does not bring us closer to effective solutions. What is needed, rather, is a well-informed biocultural approach, one that avoids both overly optimistic and overly pessimistic misconceptions, whether the goal is flying or environmental protection.

### 5.2 The possibilities of integrated bio-cultural approach

Unfortunately, the high prevalence of misuse of biological and evolutionary arguments in justifying inaction has led some scholars and even more activists to conclude that the biological viewpoints of human behavior should be better left unaddressed altogether as they could only be unhelpful or even dangerous when discussing environmental problems (e.g., Atkinson and Jacquet, 2022). Atkinson and Jacquet (2022) also commit the Moralistic Fallacy when they argue that, given it is not desirable to rationalize climate inaction, we should “challenge” all current “biological arguments” because they might “ultimately promote a reading of” essentialism, fatalism, and hopelessness, and because they were used in the past “to justify the status quo and deny the potential for social change.” It may also be tempting to deny that humans have evolved overexploitation tendencies which can be triggered under certain sociocultural conditions both in native and modern populations, in the belief that such tendencies are morally wrong or at odds with beliefs of humans as “inherently in harmony with nature.” The very position of the ecological noble savage might stem from the Moralistic Fallacy as such: since it is morally desirable that individual humans become global altruists and ultraconservationists, then human nature might only be in fact naturally inclined to sustainability. This, however, risks throwing out the baby of scientifically sound behavioral knowledge with the bathwater of the misguided and/or ideological “folk biology” and “folk psychology,” conflating evolutionary psychology with its misuse. We argue that rather than rejecting the evolutionary and biological aspects of human nature *per se* or censoring natural sciences (Jonason and Dane, 2014), what is needed is a deeper understanding and particularly greater awareness on what these evolutionary and biological aspects actually imply, a task this article has aimed to support. Even though some other recent publications have also started exposing the misuses of evolutionary explanations, e.g., in the online communities (Bachaud and Johns, 2023), there is hardly enough in-depth interdisciplinary discussion reaching beyond the polarized view of humans as either inherently doomed or ecologically noble.

When the basis of the misguided assumptions analyzed in this article are avoided, biological and evolutionary perspectives can rather start empowering us to explore innovative and more effective ways to conserve the environment, transforming psychological “barriers” into leverage points (Penn, 2003; Penn and Mysterud, 2007; Griskevicius et al., 2012; Pratarelli, 2012; Van Vugt et al., 2014; Palomo-Vélez and Van Vugt, 2021; Poškus, 2021; King and Jones, 2025). Behavioral nudges have already demonstrated some effectiveness in promoting pro-environmental behaviors by shaping choice architecture (Wee et al., 2021). For example, knowing our tendency to avoid cognitive effort, some cafeterias have started placing vegetarian dishes at the start of the cafeteria line and thus decreased customers’ meat consumption (Langen et al., 2022). They demonstrated that consumers began choosing more vegetarian food because it was easily accessible and required little cognitive effort (Langen et al., 2022). Some evolutionary researchers have also suggested that it is possible to use the evolved inclination to avoid ingesting contaminated food to invoke disgust toward meat, which may be more persuasive than health-focused appeals (Palomo-Vélez et al., 2018). Across cultures, the concerns about climate change are often influenced more by personal threats, an evolved tendency, than by “planetary threats,” which are less intuitive (Arıkan and Günay, 2021), suggesting it may be more effective to highlight the personal threats when communicating about climate change. Also status-driven motivations and kinship appeals can be used to promote green behaviors and concern for future generations (Griskevicius et al., 2012; Van Vugt et al., 2014; Palomo-Vélez and Van Vugt, 2021); for example, even though the psychological tendency of parental care may undermine sustainability in contexts where overexploitation of natural resources is perceived as necessary to provide for offspring or a privilege of the richest, the same tendency can foster pro-environmental behavior in more stable contexts, where highlighting the negative climate consequences for the development of children and grandchildren could increase the intention to adopt pro-environmental behaviors by activating intrinsic motivations for protecting one’s descendants (Van Vugt et al., 2014). Indeed, messages about the welfare of potential children increase the parental care motivation and foster ecological intentions (Palomo-Vélez et al., 2020), and responsibility to future generations significantly predicts various measures of pro-environmental behavior across political divides, which is rare (Syropoulos et al., 2025). Thus, educational campaigns emphasizing intergenerational responsibility, such as “Save the planet for your children,” can tap into this universal and deeply rooted evolutionary drive to care for one’s offspring.

Evolutionary psychology can also provide insights on how social status signaling, short-term thinking, and risk perception (Griskevicius et al., 2012; Van Vugt et al., 2014), tendencies usually conflicting ecological sustainability (Li et al., 2018, 2020; Palomo-Vélez and Van Vugt, 2021), may be turned to advantage. For example, evolutionary psychology predicts that we have evolved to seek social status and display it, because it has increased the reproductive success of our ancestors (Griskevicius et al., 2010). This tendency can be redirected toward sustainability by making eco-friendly behaviors symbols of prestige. Luxury electric cars have successfully positioned sustainability as a status symbol, encouraging high-income consumers to adopt green technologies. Similarly, companies can design sustainable products that appeal to consumers’ desire for social distinction. Mao et al. (2023) showed that hotels with GreenLeaders certification had a 6% increase in monthly revenue per available room and a 4.25% increase in their monthly average TripAdvisor rating between 2010 and 2019. They also found that the effect of GreenLeaders certification was larger for independent and upscale hotels. This suggests that by implementing “green badges” or certifications that signal environmental responsibility establishments can create a sense of upscale prestige around sustainable choices, making them more attractive to individuals. More examples of leveraging human nature to promote sustainability are discussed by King and Jones (2025).

Thus, the very human nature that can constrain pro-environmental behavior has many capacities that can also be harnessed, co-opted and repurposed to promote environmental protection (Griskevicius et al., 2012; Pratarelli, 2012; Van Vugt et al., 2014; Palomo-Vélez and Van Vugt, 2021; Poškus, 2021; King and Jones, 2025), illustrating how leveraging insights from human biology and psychology can lead to innovative and effective interventions that address challenges. As Wilson et al. (2007) put it, “the suggestion that “human nature” is a source of environmental exploitation and degradation is not a claim that nothing can be done, but a warning that effective conservation and remediation strategies will have to incorporate an understanding of relevant evolved psychological processes in order to modify human action” (p. 51; see also Wilson et al., 1998). Likewise Penn and Mysterud (2007) state that sustainability is “an admirable goal but our policies need to be sustainable themselves, and therefore we need policies that are compatible with human nature” (p. 2). Similarly, Skinner (1987) mentioned that “if human nature means the genetic endowment of the species, we cannot change it. But we have the science needed to design a world that would take that nature into account and correct many miscarriages of evolution” (p. 11). Even Atkinson and Jacquet (2022) admit that “many aspects of human psychology are flexible and contingent enough that they can be conceived as either a barrier or bridge to tackling climate change” (p. 623). As long as the interventions fit evolved inclinations and motivations to action, the conservation behaviors are far from impossible, and the better we integrate evolutionary psychology and other behavioral sciences into conservation efforts, the better equipped we are to tailor strategies to promote sustainable actions (Penn, 2003; Penn and Mysterud, 2007; Griskevicius et al., 2010, 2012; Pratarelli, 2012; Van Vugt et al., 2014; Palomo-Vélez and Van Vugt, 2021; Poškus, 2021; King and Jones, 2025).

Far from oversimplifying psychological research and its implications for climate policy as Atkinson and Jacquet (2022) suggested, the evolutionary psychological perspective actually provides novel, testable insights and underutilized pathways for pro-environmental intervention strategies that complement, rather than contradict, existing approaches. Like all psychological approaches, evolutionary-informed proposals must undergo rigorous experimental validation to determine their efficacy before scaling. The fundamental insight remains, that humans possess numerous evolved capacities that can be systematically harnessed and repurposed to address novel environmental challenges, despite having no evolutionary precedent for planetary-scale stewardship. After all, we have already done so with not just flying, but also various other modern activities from reading, writing, typing, driving, cycling, or solving complex mathematical equations, which we did certainly not evolve to perform, yet we have been able to successfully co-opt, repurpose, and integrate our evolved capacities to master these tasks after proper training (Parkinson and Wheatley, 2015). Behavioral interventions also demonstrate the power of addressing human evolved tendencies through context-specific solutions. This established pattern of biocultural innovation provides both precedent and promise for developing effective climate solutions. For instance, in Buxton, UK, a visually appealing but toxic bright blue industrial artificial lagoon posed significant safety risks to the population who desired to swim in its highly alkaline blue waters. The blank slate approach disconsidering evolved psychological inclinations presumed that simply putting warning signs would be enough. But this naive intervention strategy failed miserably as the locals continued flocking to the Bahamas-like blue toxic artificial lagoon with pH levels comparable to bleach which caused skin irritations and stomach problems among other safety risks. Finally, by dyeing the toxic water black, authorities dismantled the bright blue visual appeal which effectively deterred most visitors, showcasing how altering environmental cues to activate ancient tendencies (i.e., fear/disgust of entering dark waters) can influence behavior and reduce risk (BBC News, 2013; Wikipedia Contributors, 2025). Rather than limiting human action, the evolutionary framework highlights a broad repertoire of adaptive cultural and behavioral dispositions that can be mobilized to address pressing and unprecedented challenges (Griskevicius et al., 2010, 2012; Pratarelli, 2012; Van Vugt et al., 2014; Palomo-Vélez and Van Vugt, 2021; Poškus, 2021; King and Jones, 2025).

Still, it is crucial to acknowledge that so far, traditional behavioral interventions have yielded modest results at best. A meta-analysis encompassing over 3 million observations found that the effects of behavioral interventions aimed at promoting household action on climate change were limited, short-lived, and exhibited little enduring post-intervention impact (Nisa et al., 2019). A synthesis of 10 meta-analyses, incorporating 430 primary studies, revealed that interventions increased manifested sustainable behavior by only 2–7% compared to control groups (Bergquist et al., 2023). Aside from recycling behaviors, most household actions demonstrate low flexibility, with nudging interventions showing the largest average effect yet still yielding a mere 6.6% increase in pro-environmental behaviors in experimental groups compared to control groups (Nisa et al., 2019). Additionally, a global intervention tournament involving approximately 60 thousand participants across 63 countries found small impacts that varied by intervention type and predominantly benefited individuals who were not skeptical about climate change, while some interventions even reduced engagement in effortful climate actions, such as tree planting (Vlasceanu et al., 2024). Humans also exhibit strong habituation and resistance to changes in routine in making it difficult to sustain long-term engagement in pro-environmental actions (Nisa et al., 2019). While interventions tend to be more successful with children, their effectiveness often diminishes with age (Świątkowski et al., 2024).

Furthermore, research shows that environmental awareness, beliefs, attitudes and intentions have a very limited influence on people’s engagement in environmental actions (Hornsey et al., 2016; ElHaffar et al., 2020; Toomey, 2023; Vieira et al., 2023; see also Steg, 2018). This well-documented attitude-intention-behavior gap reveals the inadequacy of “blank slate” approaches that assume climate inaction stems primarily from knowledge deficits or incorrect beliefs. Rather, behavioral outcomes depend fundamentally on socio-ecological conditions, such as economic structures (e.g., prices, incentives, infrastructure), institutional designs, and the relative convenience of sustainable options (Whitmarsh et al., 2021). While some argue that seemingly pessimistic beliefs about human nature deterministically constrain climate action (Atkinson and Jacquet, 2022; Arbuthnott, 2025), the literature suggests these meta-beliefs are secondary to the material and structural factors that directly shape behavior (Skinner, 1987; Hornsey et al., 2016; ElHaffar et al., 2020; Whitmarsh et al., 2021; Toomey, 2023; Vieira et al., 2023). This insight redirects focus toward designing interventions that work with human nature rather than against it by creating systems where sustainable choices become the default path of least resistance, potentially bypassing the attitude-behavior gap altogether (Griskevicius et al., 2010, 2012; Pratarelli, 2012; Van Vugt et al., 2014; Palomo-Vélez and Van Vugt, 2021; Poškus, 2021; King and Jones, 2025). This recognition shifts the research priority from changing beliefs to redesigning choice architectures that automatically engage human cooperative and adaptive capacities in sustainable action.

These concerning examples of the modest effects of conventional pro-environmental interventions underscore significant limitations in the state and usage of standard psychobehavioral knowledge. Firstly, despite repeated calls for more research (e.g., Penn, 2003; Pratarelli, 2012; Van Vugt et al., 2014; Palomo-Vélez and Van Vugt, 2023), studies that adequately discuss evolved and/or biological aspects are rare (Pratarelli, 2012), particularly with regards to empirical effectiveness of behavioral interventions, and few interventions utilize the full potential of the psychological sciences for individual behavior change (Rode et al., 2024). Secondly, often due to practical matters, behavioral studies tend to concentrate on small-scale individual behaviors and nudges with limited ecological effect size, while studies on larger-scale socio-economic changes with much higher potential for large-scale behavioral changes are largely missing; while nudges may indeed tap into evolved motivations, these motivations are often activated much more strongly by economic institutions and variables (e.g., prices, income levels, monetary policies, profit-making incentives), convenient infrastructure (e.g., bike lines and availability of public transport) and other socio-economic conditions (Whitmarsh et al., 2021) which create strong pull and push mechanisms as one might also expect based on behavioral and evolutionary theory. This highlights the need to shift the focus from the inherent psychological tendencies to the ways in which these tendencies function under different socioeconomic contexts. After all, as we argued, the ecologically destructive or protective aspects of human nature are not intrinsic properties, but rather relational and contextual properties. Furthermore, rather than concentrating merely on the behaviors of consumers, evolved motivations can be studied in investors, capital owners, and policymakers which may provide much larger leeway for designing interventions which have potential to bring forth structural changes and tangible environmental impacts.

Studying evolved tendencies does not thus have to direct attention away from socio-economic factors, but rather it can integrate these factors, compare effect sizes, discover interactions, and thus disentangle which interventions are, in real life, having considerable ecological effect sizes. Interdisciplinary research that bridges cultural and biological evolution is urgently needed (Ehrlich, 2002; Richerson et al., 2024) as we clearly still lack a realistic image and awareness of the complex biosocial drivers behind environmental problems, calling for tighter collaboration, capacity-building and openness between evolutionary psychologists and social scientists as well as education efforts of public and media. It may be particularly useful to study which communication methods would best mitigate the risk of the misuse of evolutionary and biological arguments to prevent inadvertently triggering essentialist interpretations. Journalists and academics in the humanities and social sciences could also benefit from familiarizing with modern biology to diminish the risks of succumbing to essentialist biases (Dar-Nimrod et al., 2021).

Moral considerations naturally play a crucial role when designing studies which address evolved and/or biological aspects of human behavior, as in any field of research. Green nudges need to go through the same rigorous ethical processes (including open design and democratic acceptance), as nudges or policies in general to avoid manipulative and invasive actions which would cause distrust and resistance among target audiences (Nisa et al., 2019). And regardless of the underlying drivers of human behaviors, it can be argued that we have a moral duty to preserve the environment and promote sustainable development (Cripps, 2013) as emphasized by key international frameworks such as the Stockholm Declaration (1972), Rio Declaration (1992), the 2030 Agenda for Sustainable Development (United Nations, 2015) and the Declaration of Ethical Principles in Relation to Climate Change (Unesco, 2017). Moreover, even if the “ecologically noble savage” concept is a romanticized oversimplification, this should not be used for depoliticizing the humanistic issue concerning the rights of native peoples, excusing the invasion and destruction of their land and natural resources by extractivist companies, such as logging and mining, nor undermining the United Nations Declaration on the Rights of Indigenous Peoples (United Nations General Assembly, 2007). Furthermore, the focus on human nature and psychological approach to sustainability should not distract nor undermine the macro societal approach in dealing with to the major contributors to environmental crisis: oil corporations (Heede, 2014; Bonneuil et al., 2021) and wealthy individuals (Kenner, 2019; Chancel, 2022), Appeals to nature and biology have been repeatedly used in history to justify the status quo, slavery, racism, sexism, and other morally questionable practices, but this does not have to mean that modern biology should be suppressed or denied (Pinker, 2003); rather, what is needed is vigilance and robust regulations to prevent unethical misapplications while fostering accurate understanding of behavioral sciences and responsible journalism. One might draw a parallel to flying again: aeroplanes are also used for terrorist attacks, yet rather than prohibiting flights altogether, we have taken up various safety and oversight measures to avoid misuse.

When pondering over biological determinism, it is good to remember that Earth is inhabited by millions of species which have all evolved following the same apparently “selfish” principles of natural selection, yet, only few of those species exhibit clear overexploitative patterns, and it is still debated which factors prevent populations from consuming themselves to extinction (Vuorinen et al., 2021; Gutiérrez Al-Khudhairy and Rossberg, 2022; Oksanen et al., 2023). Rather than asking why any life form might be inherently doomed to destroying its environment, it is more important to ask: under what conditions is it *not*? And with regards to humans, evolutionary psychology and other behavioral sciences are well placed to answer this question. If we better understand the complex biocultural nature of our species, perhaps, one day, we will be able to implement the changes required for enabling effective environmental behaviors to take off toward sustainability. Overcoming inaction leveraging human nature requires avoiding misconceptions and promoting a nuanced and integrative perspective. Transforming psychological “barriers” into leverage points offers a promising path to promote effective, scalable, and lasting actions for planetary protection (Griskevicius et al., 2010, 2012; Pratarelli, 2012; Van Vugt et al., 2014; Palomo-Vélez and Van Vugt, 2021; Poškus, 2021; King and Jones, 2025). Continued investigation of these dynamics will be essential in navigating the complexities of the ecological challenges ahead, while understanding our evolved psychology is crucial for effectively addressing the ecoclimatic polycrisis.

## 6 Conclusion

Our analysis makes three fundamental contributions to the sustainability sciences. First, we have deconstructed the false dichotomy between romanticized and fatalistic views of human nature, demonstrating for instance how both positions along a continuum commit analogous category errors by treating sustainability as an inherent trait rather than a context-dependent outcome. Sustainability is neither guaranteed nor precluded by human nature, but shaped by integrative systems that align human flexibility with ecological constraints. Second, we have advanced a biocultural framework that reconciles evolutionary psychology with behavioral plasticity, showing how human capacities for innovation, cooperation, interdependence, communication and self-regulation can be systematically engaged to possibly overcome climate inaction and decrease the current environmental crisis. Such an approach better captures human nature’s variability and plasticity, acknowledging that environmental outcomes depend on how cultural, economic, and institutional contexts modulates the activation and inactivation of specific sets of evolved capacities. Third, we have charted a path forward for intervention science by identifying a few key leverage points where socioecological systems interact with our evolved psychology, such as economic incentives, choice architectures, and institutional designs, that can transform sustainability from a mere educational challenge into the emergent product of properly structured human environments.

Rather than debating whether beliefs of human nature helps or hinders environmental action per se, we call for rigorous interdisciplinary research programs that test how to optimally align our species’ unique biocultural adaptability with the unprecedented demands of planetary stewardship. Evolved, universal psychological tendencies neither justify inaction nor make sustainability unattainable, instead, correctly understanding human nature serves as a crucial foundation for guiding us toward designing effective and lasting sustainable practices. The pressing question is no longer what humans inherently are, but how to create the conditions that best elicit what we can become to save the planet.

We have gone a long way to promote a much-needed fundamental update and reassessment of how evolutionary perspectives on sustainability are ultimately depicted, understood and used. Evolutionary perspectives need to be properly assessed to fulfill its potential in informing and advancing sustainability sciences. This necessarily involves rejecting outdated and misguided impressions in favor of frameworks grounded in contemporary evolutionary psychological and behavioral sciences. Our proposed shift from dispositionist to interactionist, deterministic to probabilistic, fixist to plastic, essentialist to populationist and contextualistic, pessimist to realist, and from fatalistic to possibilistic corrects historical miscomprehensions. Our synthesis demonstrated and recognized human behavior as neither inherently virtuous nor overexploitative, but dynamically and predictably responsive to incentives, norms, sociocultural institutions, and socio-ecological restrictions. This evolutionary perspective is both scientifically robust and pragmatically hopeful, as it provides a promising framework empowering researchers to design interventions that work with, rather than against human nature, systematically engaging humanity’s cooperative, regulative, and adaptive potential to promote a sustainable common future. We hope to inform researchers outside the field of evolutionary psychological sciences about the actual content and the importance of the evolutionary perspective to offer new horizons in dealing with the ecoclimate crisis.

## Footnotes

^1^Alternative splicing is a cellular process by which different messenger RNAs are produced from the same primary transcript, through variations in the splicing pattern of the transcript. Thus, the same gene can ultimately give rise to different proteins (Griffiths et al., 2012).

^2^Epigenetics are nongenetic reversible chemical changes in histones or DNA that alter gene function, turning genes on or off for gene expression, without altering the DNA sequence. Thus, having a gene does not guarantee that it will always be expressed (Griffiths et al., 2012).

^3^Incomplete penetrance occurs when not every individual with a given genotype expresses the corresponding phenotype because of other genes or environmental factors. Thus, having a combination of genes does not necessarily lead to the associated phenotypic expression (Griffiths et al., 2012).

^4^Variable expressivity occurs when individuals with the same genotype exhibit different degrees of a trait’s expression. Thus, even when a genetic predisposition is expressed phenotypically, it can occur with varying intensities in different individuals (Griffiths et al., 2012).

^5^Plasticity refers to the organism’s evolved ability to change its cognition and behavior in response to environmental demands (Bjorklund, 2016).

^6^Ontogenesis refers to how a particular behavioral pattern changes during an organism’s life span. Both genetic and environmental processes influence the modification of the regulatory mechanisms that produce behavior during the individual development (Tinbergen, 1963).

^7^Acclimatization refers to a longer-term, reversible and coordinated adjustment to multiple environmental factors or stressors in a natural environment (Collier et al., 2019).

^8^Population thinking is defined as “a methodological doctrine stating that regularities that occur in populations such as extinction, speciation, and adaptation emerge from the collective activities of individuals” (Ariew, 2009).
